# Is the trend of increasing use of patient-reported outcome measures in medical device studies the sign of shift towards value-based purchasing in Europe?

**DOI:** 10.1007/s10198-019-01070-1

**Published:** 2019-05-16

**Authors:** Miklós Weszl, Fanni Rencz, Valentin Brodszky

**Affiliations:** 1 Department of Health Economics, Corvinus University of Budapest, Fővám tér 8, Budapest, H-1093 Hungary; 2 Premium Postdoctoral Research Program, Hungarian Academy of Sciences, Nádor u. 7, Budapest, H-1051 Hungary

**Keywords:** Medical device, Patient-reported outcome, Value-based purchasing, Europe, I10

## Abstract

**Background:**

The recent update of the European Union’s (EU) regulation on public procurement has created new opportunity for progress in the purchasing of medical devices by shifting towards focus on value from one purely on price. Patient-reported outcome measures (PROMs) may serve as additional tools for manufacturers to demonstrate value beyond traditional metrics of safety and performance and to differentiate their products in a market of increasing competition. The aim of our study was to investigate the extent to which PROMs are included in registered device studies in the EU and interpret the results in the context of the purchasing of medical devices.

**Methods:**

Twelve device groups were searched in clinical trial registries to determine the frequency distribution of PROMs in related studies.

**Results:**

Results indicate that clinical studies of the selected device categories are done predominately in the western EU nations and are increasingly including PROMs. In the United Kingdom 121 (65%) study, out of 186 included PROMs, and in Germany, 92 (52%) out of 178 between 1998 and 2018. Few device studies were done in the Central and Eastern European region, and out of 76 studies 27 (35%) included PROMs. Since there is no requirement to include PROMs in device studies for regulatory purposes, it seems probable that their increasing use is driven by competitive market pressures.

**Conclusion:**

The trend of increasing use of PROMs might be driven by the demand of purchasers to demonstrate value of devices, but is manifested at different levels in various regions of the EU.

## Introduction

The role of medical devices has dramatically increased in the provision of healthcare services in the last few decades. Access to state-of-the-art devices allows the diagnosis and treatment of diseases that have not been detectable and/or treatable earlier. Medical device manufacturers need to differentiate their products on a highly competitive market. The payers, including hospitals and health insurance entities, make cost-conscious purchasing decisions to maximize the effectiveness of healthcare services. Device manufacturers need to demonstrate product value beyond the traditional measures of clinical safety and efficacy. Patient-reported outcomes (PROs) may be suitable for this purpose [1].

PRO measures (PROMs) the perception of patients on their own experience and outcomes, including health-related quality of life, in response to an intervention. These measures can complement other clinical and physiological information or, in some cases, substitute for them [2]. In addition, PROMs may allow gathering important information on health status that is not detectable by other measures [3]. For instance, in orthopedic studies, PROMs are commonly used to quantify functional mobility, pain levels, and disability [4]. Thus, PROMs can serve as components in composite endpoints for clinical studies [5].

In the United States between 2009 and 2015, a > 500% increase was observed in the number of device studies that included PROMs [6]. It was also observed that the PROMs were often based on subjectively collected data with the potential for unreliable measurement [7]. The US Food and Drug Administration (FDA) launched the Medical Device Development Tool Program in 2017 to assist study sponsors to incorporate valid PROMs into device clinical trials [8].

Different stakeholders have different perspectives on PROMs. Regulatory agencies, like FDA in the United States and Notified Bodies in the European Union, may use PROMs to support or deny the claim of the manufacturer concerning the safety or efficiency of a device. Payers and providers may use PROMs to compare alternative devices, including new devices with modest design modification in comparison with previous versions, for purposes of both purchasing and clinical implementation [9, 10].

There is an ethical obligation to disclose the findings of clinical trials. Only half of the studies of high-risk medical devices are published and there often are discrepancies in important study features between the summary provided to the regulatory agencies and the eventual publication in peer-reviewed journals [11, 12]. The extent of publication bias is measurable in those countries, where clinical trial registries provide information on the device studies in accordance with the requirements of the International Committee of Medical Journal Editors (IMCJE) [13]. The European Clinical Trial Register, however, does not provide information on clinical trials for medical devices [14]. Although every member states of the European Union (EU) require the registration of device clinical studies before commencing studies, there are considerable differences in the publicly available information on study features. The German Clinical Trials Register (DRKS), the United Kingdom’s ISRCTN Registry, ClinicalTrials.gov, and the International Clinical Trials Registry Platform (ICTRP) of the World Health Organization make important details available for the public. In contrast, most of the member states of the EU do not have clinical trial registries with that level of transparency. Data from these registries may provide more accurate estimates of the prevalence of PRO measures used in clinical trials for medical devices than a review of peer-reviewed journals.

The aim of this paper is to show the trend in the use of PROMs for registered device studies in the EU and interpret the results in the context of the purchasing of medical devices. Owing to the diversity of medical devices, our study was limited to twelve specific device categories and we attempted to extrapolate to the overall role of PROMs in the procurement of medical devices in the EU.

## Methods

### Selection of medical devices

Twelve implantable and high-risk (class IIb & III) medical device therapeutic groups were selected for investigation according to the risk-based classification system of the Medical Device Regulation 2017/745. Six out of the 12 were class IIb devices, including dental implants, peripheral venous catheters, intramedullary nails, orthopedic plates, spinal fusion devices, and contact lens solutions. The other six devices belong to class III risk category, including cardiac pacemakers, bone grafts, hip implants, central venous catheters, intraocular lens, and absorbable sutures. Most of the selected device groups are intended for cardiologic and orthopedic interventions that are among the most expensive and risky service lines [15]. The other device groups were selected based on the large populations using them.

### Search of clinical trial databases

The German Clinical Trials Register, the United Kingdom’s ISRCTN Registry, ClinicalTrials.gov, and the International Clinical Trials Registry Platform of the World Health Organization were searched manually to identify clinical trials of the 12 categories of medical devices. The search was restricted to studies that have been conducted in the EU. The database search was carried until December 31, 2018. Interventional and observational studies were searched without limitation on time period. Completed, active, terminated, and studies in recruiting phase were included in the search. The search strategy was constructed using a single set of keywords, such terms identifying medical device categories: dental implant OR hip implant OR orthopedic plate OR cardiac pacemaker OR intramedullary nail OR absorbable suture OR spinal fusion device OR bone graft OR contact lens solution OR intra-ocular lens OR peripheral venous catheter OR central venous catheter. No further limitation was applied. All search hits were assessed without exclusion. Duplications were excluded by marking each hit in the databases after the review of the belonging synopsis. Studies were taken into consideration for further analysis if they disclosed clear information on (i) the objective of the study, (ii) test device, (iii) the experimental design, (iv) endpoints, and (v) the sponsor. Studies were excluded from further analysis if they were designed to assess the efficiency/effectiveness of surgical or treatment approaches in general, or if the applied device was not clearly identifiable. The quality of the published synopses was not evaluated in terms of comprehensiveness and accuracy. All PROMs used in device studies either as primary or secondary outcomes were recorded for each device categories along with the starting date and locations of the studies.

### Data analysis

Clinical studies of medical devices were counted for each year until 2018, and the distribution of the studies was determined between the member states of the EU. Multicenter studies were taken into account in each country, where they had investigational site, as if they were local, single-center studies. The proportion of the most often used health-related quality-of-life (HRQoL) and disease-specific outcome measures was determined for all selected device groups. The number and proportion of studies that included PROMs were measured, as were trends over time.

## Results

**Fig. 1:**
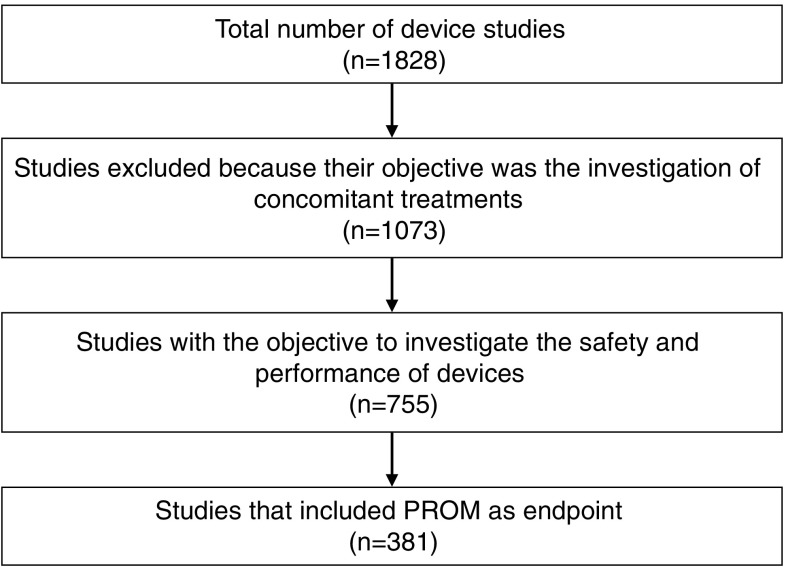
Methodology and result of clinical trial registry search

The first device studies were registered in 1998 in the searched databases. Altogether, 1,828 publicly available synopses of clinical studies were reviewed in relation to the selected device categories concerning the time period from 1998 to 2018. Of these, 1073 were excluded from further analysis, because their objective was not the investigation of the clinical safety or performance of devices but, rather, that of concomitant interventions. Of the remaining 755 device studies, 381 (50%) included PROMs as primary or secondary endpoints, implying single and multicenter studies (Fig. 1).

**Fig. 2:**
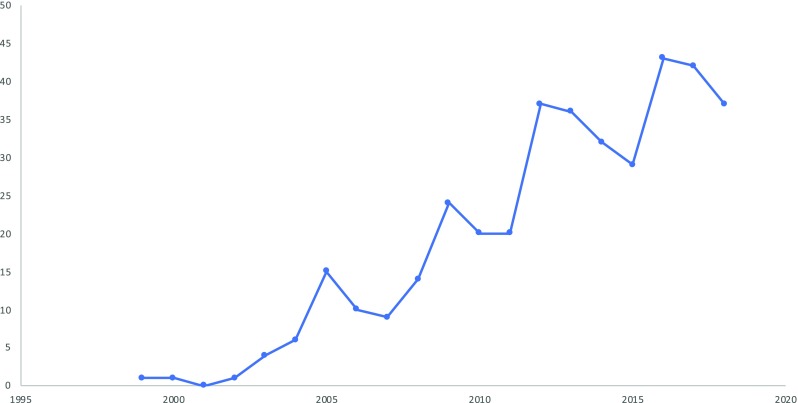
Increasing trend in the quantity of clinical studies of medical devices that included PROM as primary or secondary endpoints

Our results show that an increasing proportion of medical device studies include PROMs in the EU (Fig. 2). Almost all (94%) of these studies were conducted in Western European countries, with the United Kingdom (24%) and Germany (18%) standing out. Only 6% of the studies were performed in the Central Eastern European (CEE) region, the majority of them in Poland and Hungary, respectively. PROMs were not included at all in device studies in Latvia, Lithuania, and Estonia (Table 1). Out of the 381 device, studies that included PROMs 48 (12.6%) were multicenter studies, i.e., 14 (12.6%) for hip implants, 4 (10.5%) for bone grafts, 3 (7.14%) for cardiac pacemakers, 6 (17.1%) for intraocular lens, 11 (27.5%) for spinal fusion devices, 5 (17.8) for intramedullary nails, and 5 (10.4%) for orthopedic plates.

**Table 1:** Distribution of studies of selected device categories between the EU member states

EU member states	Total number of studies		Number of studies that included PRO measures		Proportion of studies included PROM (%)
United Kingdom	186	(18%)	121	(24%)	65
Germany	178	(17%)	92	(18%)	52
France	108	(10%)	42	(10%)	39
Italy	98	(9.5%)	16	(3%)	16
Spain	79	(7.7%)	39	(8%)	49
The Netherlands	65	(6.3%)	28	(5%)	43
Belgium	57	(5.5%)	28	(5%)	49
Austria	56	(5.5%)	13	(2.5%)	23
Denmark	54	(5.2%)	27	(5%)	50
Sweden	52	(5%)	27	(5%)	52
Switzerland	52	(5%)	13	(2.5%)	25
Norway	28	(2.7%)	25	(5%)	89
Poland	23	(2.2%	9	(2%)	39
Finland	18	(1.7%)	5	(1%)	28
Hungary	18	(1.7%)	4	(0.8%)	22
Czech Republic	14	(1.4%)	5	(1%)	36
Portugal	14	(1.4%)	5	(1%)	36
Slovakia	7	(0.7%)	4	(0.8%)	57
Greece	4	(0.4%)	3	(0.6%)	75
Ireland	4	(0.4%)	2	(0.4%)	50
Latvia	4	(0.4%)	0	(0%)	0
Romania	4	(0.4%)	3	(0.6%)	75
Lithuania	3	(0.3%)	0	(0%)	0
Croatia	1	(0.1%)	1	(0.2%)	100
Estonia	1	(0.1%)	0	(0%)	0
Slovenia	1	(0.1%)	1	(0.2%)	100

Concerning the CEE region, 27 (35%) device studies included some kind of PROMs. Most of the device studies are organized in Poland and in Hungary, respectively. There is no data on device related clinical studies from Bulgaria. CEE region implies Poland, Czech Republic, Slovakia, Hungary, Slovenia, Croatia, Romania, Bulgaria and the Baltic states

Concerning the selected device categories, most of the trauma and orthopedic device studies included PROMs, whereas they were used significantly less frequently in other device studies (Table 2). PROMs were included in 46 (48%) studies of orthopedic plates, in 103 (44%) hip implant, 36 (32%) spinal fusion, 26 (43%) intramedullary nail, 12 (22%) absorbable suture, 33 (15%) bone graft, 33 (15%) intraocular lens, 9 (22%) contact lens solution, 20 (10%) dental implant, 50 (15%) cardiac pacemaker, 33 (57%) peripheral venous catheter, and in 10 (5%) central venous catheter studies.

**Table 2:** Frequency distribution of device studies with and without PROMs

Device categories	Total number of studies	Number of studies included PROM	Proportion of studies included PROM (%)
Absorbable sutures	55	12	22
Bone grafts	228	33	15
Cardiac pacemakers	339	50	15
Central venous catheters	182	10	5
Contact lens solutions	40	9	22
Dental implants	202	20	10
Hip implants	236	103	44
Intramedullary nails	61	26	43
Intraocular lens	218	33	15
Orthopedic plates	95	46	48
Peripheral venous catheters	58	33	57
Spinal fusion devices	114	36	32

PROMs were included in orthopedic and trauma device studies more often than in other device categories

The frequency of general HRQoL measures in device studies is shown in Table 3. General HRQoL measures, such as EQ-5D (29.7%), Short Form 36 (SF-36) (6.6%), Short Form 12 (SF-12) (2.6%), and patient satisfaction index (PSI) (1%) turned up in the reviewed device studies. Pain associated with interventions was often measured on visual analogue scale (VAS) (19.7%), but numeric-rating scale (NRS) (0.3%) appeared in our search, as well (Table 3). In the CEE region, 76 device studies were carried out and 27 (35%) of them included PROMs. Out of the 27, VAS was applied in 12 studies (44%), EQ-5D in 10 studies (32%), SF-36 in 7 studies (26%), ODI in 6 studies (22%), SF-08 in 2 studies (7%), HHS in 2 studies (7%), OHS in 1 study (4%), and NRS in 1 study (4%).

**Table 3:** Frequency of general HRQoL measures in device studies

General HRQoL measures	Frequency in relation to the number of studies included PROM (%)	Frequency in relation to the total number of device studies (%)
EQ-5D	29.7	6.2
VAS	19.7	4.1
SF-36	6.6	1.4
SF-12	2.6	0.5
PSI	1.0	0.2
NRS	0.3	0.1
Total	59.9	12.5

General HRQoL measures are still relatively rarely used (12.5%) in device studies. In studies that included PROMs general HRQoL measures were applied in 59.9%. Out of the six identified measures, EQ-5D is the dominant followed by VAS, while the frequency of other measures is far behind these two

Disease-specific PROMs showed a large diversity in trauma and orthopedic device studies. Minnesota Living with Heart Failure (MLWHF) was the only cardiac disease-specific PRO instrument that turned up in conjunction with cardiac pacemaker studies (Table 4). Regarding the selected orthopedic and trauma devices, osteoarthritis (WOMAC and HOOS), pain, and function related to total hip arthroplasty (HOS), functionality of upper extremities (DASH), disability (ODI), and pain in the wrist joint (PWRE) measures were most often applied. Harris Hip Score (HHS), which is a composite measure of clinician-, and patient-reported outcomes that encompasses subscales for the function and pain in relation to the hip disorders, also was used frequently. For ophthalmological devices, vision-targeted health status (VFQ25), visual function index (VF-14), and the contact lens user experience (CLUE) were used most frequently. Concerning dental implants, oral health impact profile (OHIP) has been applied to measure the oral health-related quality of life of patient after dental implant surgery (Table 4). In a number of cases, the intention to apply a PROM was clearly indicated in the synopses of the device studies, but the actual instrument was not defined (data not shown).

**Table 4:** Frequency of general and disease-specific PROMs in device studies

Device categories	Frequency of general HRQoL measures (% of total studies/% of studies with PROM)	Frequency of disease-specific PROMs (% of total studies/in % of studies with PROM)	Proportion of general HRQoL measures (to the total number of studies/to studies with PROM)
Absorbable sutures	VAS (2%/17%)	–	NA
Bone grafts	VAS (7%/48%) EQ-5D (3%/21%) SF-36 (2%/12%) SF-12 (2%/12%)	ODI (1%/9%)	0.1 0.9
Cardiac pacemakers	EQ-5D (2%/12%) SF-36 (2%/16%) VAS (1%/10%)	MLWHF (3%/20%)	0.06 0,4
Central venous catheters	SF-36 (1%/20%) VAS (1%/20%) EQ-5D (0.5%/10%)	QLQC30 (1%/20%) VAD (0.5%/10%) QASICC (0.5%/10%)	0.3 0.5
Contact lens solutions	VAS (3%/11%)	CLUE (1%/11%) VFQ25 (1%/11%)	0.025 0.1
Dental implants	VAS (2.5%/25%)	OHIP-14 (1%/10%) OHIP-15 (0.5%/5%) OHIP-G-49 (0.5%/5%)	0.05 0.25
Hip implants	EQ-5D (21%/46%)	HHS (28%/63%) OHS (18%/40%) WOMAC (7%/15%) HOOS (7%/15%)	0.2 0.5
Intramedullary nails	EQ-5D (16%/38%) VAS (8%/19%)	HHS (8%/19%) DASH (8%/19%)	0.25 0.6
Intraocular lens	EQ-5D (1%/6%) VAS (1%/6%)	VF-14 (2%/15%) VFQ25 (2%/12%)	0.02 0.1
Orthopedic plates	EQ-5D (35%/72%) VAS (12%/24%) SF-12 (4%/9%)	DASH (11%/22%) PRWE (6%/13%)	0.5 1.1
Peripheral venous catheters	VAS (3%/67%) NRS (2%/33%)	–	NA
Spinal fusion devices	VAS (23%/67%) SF-36 (10%/28%) EQ-5D (9%/11%) PSI (4%/6%)	ODI (21%/31%)	0.4 1.4

In the first column, the general health-related quality-of-life (HRQoL) measures are set out. In the brackets, the first value expressed in percentage indicates the frequency of PROMs in relation to the total number of studies in the concerned device category. The second percentage indicates the frequency of the PROM concerned relative to the number of studies that included PROMs. In the second column, the frequency of disease-specific PROMs is indicated according to the same methodology. The third column indicates the proportion of HRQoL measures relative to the total number of studies (upper value) and to the overall number of studies that included PROMs in the concerned device category. Values close to 0 indicate rare use of general HRQoL measures in the studies, while values close to 1 mean most of the studies included at least one of measure. Values over 1 means that studies included more than one general HRQoL measures in average. The use of general HRQoL measures was the highest in clinical studies of spinal fusion devices, orthopedic plates, and bone grafts, respectively

E*Q-5D* EuroQol-5-dimensions, *VAS* visual analogue scale, *SF-08/36/12* short form 36/12, *MLWHF* Minnesota Living with Heart Failure, *ODI* Oswestry disability index, *HHS* Harris hip score, *OHS* Oxford hip score, *WOMAC* Western Ontario and McMaster universities osteoarthritis index, *HOOS* hip disability and osteoarthritis outcome score, *VFQ-25* visual functioning questionnaire, *NRS* numeric-rating scale, *CLUE* contact lens user experience, *PSI* patient satisfaction index, *DASH* disabilities of the arm, shoulder and hand, *OHIP* oral health impact profile, *PRWE* patient-rated wrist evaluation

Two-thirds (68%) of the studies that included PROMs were sponsored by research organizations or by government, with the remainder sponsored by industry. In 14% of the research organization-sponsored studies, the device manufacturer participated either as a collaborator or as the funding party.

## Discussion

There is a strong trend towards increased use of PROMs in clinical studies of medical devices, most of which are conducted in the Western European region. General HRQoL measures turned up in each device category, though their use is quite rare compared to pharmaceutical studies [10]. Disease-specific instruments were used most frequently in orthopedic and trauma related studies, providing more accurate and detailed information about patient’s experience. In terms of sponsorship, the distribution of studies that included PROMs was similar between research organizations and industry.

The increasing use of PROMs in device studies in the EU is comparable to that in the United States. The studies in the United States were all linked to label claims granted by the FDA [6]. In contrast, the industry-sponsored studies in our research might not be construed as pilot or pivotal studies to support the Conformité européenne (CE) marking of products. Most of the reviewed studies had no publicly available results in the searched clinical trial registries, perhaps because not all device studies are conducted for regulatory purposes.

For CE marking, the acceptability of a medical device is based on comparison to existing alternatives. To avoid pre-market clinical studies, manufacturers often claim equivalence with CE-marked devices that are already in clinical use. This has kept the regulatory barrier low for medical devices, in comparison with the relatively high regulatory barriers for drugs. Pre-market clinical investigations are required for the CE marking of implantable and high-risk medical devices of new design.

The increasing number of device studies and the inclusion of PROMs may be attributed to the recent EU directive (Directive 2014/24/EU) on public procurement. Public procurement is the acquisition of goods and services on behalf of a public authority or other body, such as hospital or other healthcare provider. In the EU, a large fraction of medical devices is purchased through public procurement, including consumable and implantable devices [16]. The public procurements are increasingly relying on auction mechanism, which starts with the publication of the requirements of the contracting body. Expressions of interest then are invited from potential tenderers [17]. The second step involves the invitation to pre-selected tenderers who have the requisite level of professional, technical, and financial expertise and capacity. The submitted tenders are evaluated according to their compliance with the published award criteria that may be either the lowest price or the most economically advantageous tender (MEAT). The lowest price evaluation method is the most transparent, but quality may only be taken into account to the extent of the upfront fixed technical specification. The MEAT method allows the evaluation of both price and quality, including technical merits and functional characteristics, which requires technical competence on the part of the tenderer [18]. The public procurement of medical technologies is often decentralized at hospital level, because only the end-user clinicians are able to evaluate the possible clinical outcome of a device.

The new regulatory framework was meant to shift procurement from the lowest price approach towards a value-based approach by encouraging public authorities to put more emphasis on quality, life-cycle costing, cost effectiveness, and societal benefits. A recent report shows that Sweden, the United Kingdom, Germany, Spain, France, Switzerland, Italy, and The Netherlands have incorporated the directive into their own national law. Six core categories of medical devices are moving towards value-based procurement, albeit at different speeds. The report suggests that orthopedics and cardiology are expected to be the most advanced in the transition over the next few years [19]. Our results support this assumption as trauma and orthopedic device studies included PROMs the most often, though this does not hold completely true for cardiologic devices. Based on these findings, we speculate that the growth in use of PROMs has been localized in countries that have started to shift their public procurement from price towards value-based (price and quality) approach [20]. For commodity products, such as absorbable sutures, catheters, and contact lens solutions, price may remain the most important procurement criterion. Our results coincide with this assumption as well, since PROMs were considerably less often included in studies of these device categories. In conclusion, the trend of volunteer inclusion of PROMs in clinical studies of some particular medical devices, which are used in the most expensive and risky service lines may be a sign of the increasing demand by purchasers for the demonstration of safety and performance in addition to price.

The results of our study should be interpreted in light of its limitations. Since information listed in clinical trial registries relies on study sponsors, we cannot be certain that the data are current and accurate. Not all device studies may have been registered in the searched databases. The limitation of our research to 12 device groups limits generalizability. For instance, cardiologic devices were supposed to include PROMs in comparable quantity to trauma and orthopedic device studies. The random selection of only a few cardiologic devices could cause selection bias that reduces the reliability of the results. The variability of applied PROMs concerning the various device categories may arise from the disparity in the existence of suitable instruments.
